# Whole-body magnetic resonance imaging for detection of skeletal metastases in children and young people with primary solid tumors - systematic review

**DOI:** 10.1007/s00247-017-4013-8

**Published:** 2017-11-18

**Authors:** A. M. Smets, E. E. Deurloo, T. J. E. Slager, J. Stoker, S. Bipat

**Affiliations:** Department of Radiology and Nuclear Medicine, Academic Medical Center (AMC), Meibergdreef 9, 1105 AZ Amsterdam, the Netherlands

**Keywords:** Adolescents, Bone, Cancer, Children, Metastasis, Solid tumors, Whole-body magnetic resonance imaging, Young adults

## Abstract

**Background:**

Many solid neoplasms have a propensity for osteomedullary metastases of which detection is important for staging and subsequent treatment. Whole-body magnetic resonance imaging (WB-MRI) has been shown to accurately detect osteomedullary metastases in adults, but these findings cannot be unconditionally extrapolated to staging of children with malignant solid tumors.

**Objective:**

To conduct a literature review on the sensitivity of WB-MRI for detecting skeletal metastases in children with solid tumors.

**Materials and methods:**

Searches in MEDLINE and EMBASE databases up to 15 May 2017 were performed to identify studies on the diagnostic value of WB-MRI. Inclusion criteria were children and adolescents (age <21 years) with a primary solid tumor who were evaluated for skeletal metastases by WB-MRI and compared to any type of reference standard. The number of included patients had to be at least five and data on true positives, true negatives, false-positives and false-negatives had to be extractable.

**Results:**

Five studies including 132 patients (96 patients with solid tumors) were eligible. Patient groups and used reference tests were heterogeneous, producing unclear or high risk of bias. Sensitivity of WB-MRI ranged between 82% and 100%. The positive predictive value of WB-MRI was variable among the studies and influenced by the used reference standard.

**Conclusion:**

Although WB-MRI may seem a promising radiation-free technique for the detection of skeletal metastases in children with solid tumors, published studies are small and too heterogeneous to provide conclusive evidence that WB-MRI can be an alternative to currently used imaging techniques.

**Electronic supplementary material:**

The online version of this article (10.1007/s00247-017-4013-8) contains supplementary material, which is available to authorized users.

## Introduction

Children with malignant solid tumors may present with skeletal metastases at diagnosis with a varying frequency according to the type of tumor. More than 50% of patients with neuroblastoma and up to 6% of patients with rhabdomyosarcoma have metastases in bone marrow and/or cortical bone at diagnosis [1–3]. Patients with Ewing sarcoma have bone metastases (with or without lung metastases) at diagnosis in up to 11.5% [4]. Osteosarcoma patients sometimes present with skip lesions, i.e. bone metastases within the same bone or across a neighboring joint or with bone metastases at diagnosis [5, 6] and a rare pediatric renal tumor, clear cell sarcoma of the kidney, is known for its propensity to metastasize to bone [7]. Accurately detecting bone and bone marrow metastases is essential for treatment assignment and prognosis. Conventional radiography, computed tomography (CT), and various scintigraphic techniques (bone scan, 123I meta-iodobenzylguanidine scintigraphy [MIBG], 18F–fluorodeoxyglucose positron emission tomography [FDG-PET]), are currently used for staging, yet all these techniques expose the patient to ionizing radiation and have a variable sensitivity for detecting osteomedullary metastases in different types of tumors [8–13]. Consequently, there is an increasing interest in alternative, preferably radiation-free, techniques with a track record in osteomedullary pathology, such as whole-body magnetic resonance imaging (WB-MRI) [14].

Several studies have shown WB-MRI to be useful in evaluating adult cancer patients [15–21] and MRI to be a sensitive technique for detecting osteomedullary lesions in children [22, 23]. Publications on the usefulness of WB-MRI in pediatric cancer patients are still limited; only a few studies have investigated the potential of WB-MRI for detecting skeletal metastases specifically in children [24–28]. The difference in tumor types and the complexity of bone marrow conversion from red to yellow marrow that takes place during childhood and adolescence call for evidence on the value of WB-MRI in pediatric oncology staging specifically.

The purpose of this study was to assess the diagnostic value of WB-MRI for detecting skeletal metastases in children and adolescents with primary solid tumors as judged from the available literature.

## Materials and methods

### Search strategy

Computerized searches in MEDLINE and EMBASE databases until 15 May 2017 were performed to identify studies reporting on the diagnostic value of WB-MRI in children and young people (ages <21 years) with primary solid tumors. The literature search consisted of terms related to the three main elements: 1. Whole-Body Magnetic Resonance Imaging and 2. Patient population (oncological patients) and 3. Childhood/Adolescence. Different terms were used to have a broad search to identify all relevant studies (Supplement 1).

### Selection of relevant studies

The selection of relevant studies was performed by one author with experience in data extraction of 25 meta-analyses (SB, Clinical epidemiologist with 14 years expertise in evidence based radiology).

Step 1: The title, article type and abstract of all retrieved articles were checked for potential relevance. Duplicates (i.e. identical articles found in both databases) and non relevant articles, such as letters, comments, editorials and conference papers, that were not disease related and other imaging were excluded and remaining articles were considered potentially relevant.
Step 2: All potentially relevant articles were checked on full text by the same author for further selection. During this step, reviews, studies on other outcomes, case reports and studies on adults were excluded. After an interval of 2 weeks, the author double-checked this step to make sure all relevant papers were selected for further analyses.

### Inclusion criteria

All remaining articles were considered relevant and full text was checked on inclusion criteria by two pediatric radiologists (AMJBS, 25 years experience in pediatric oncological imaging and EED, 8 years experience in pediatric oncological imaging) with 25 and 8 years of experience, respectively, in pediatric oncological imaging.

Inclusion criteria were:

1. Children or adolescents (up to 21 years) with any of the following primary solid tumors: neuroblastoma, soft-tissue sarcoma, Ewing sarcoma, osteosarcoma, hepatoblastoma or renal tumors. If both children/adolescents and adults were included, at least 90% of the study population had to be children/adolescents or data on children/adolescents could be separately extracted. If data were combined with other types of tumors, e.g., lymphoma, data on primary solid tumors could be extracted separately;
2. Number of patients ≥5;
3. Evaluation of WB-MRI compared with any type of reference standard (such as MIBG-, bone marrow biopsy, bone biopsy trephine, FDG-PET, CT, bone scan, etc.);
4. Evaluation of skeletal metastases by WB-MRI (if other types of metastases [extra skeletal] were included, at least 75% had to be skeletal metastases or data on skeletal metastases could be extracted separately);
5. Data on true positives, true negatives, false-positives and false-negatives could be extracted.

### Data extraction of included papers

Data extraction was also performed by AMJBS and EED by using a standardized form. The following data were assessed: 1. Methodological quality characteristics; 2. Study design characteristics; 3. Patient characteristics; 4. Technical characteristics regarding WB-MRI; 5. Interpretation of WB-MRI, 6. Reference standard and flow of timing and 7. Data on accuracy. Disagreement was resolved through discussion amongst SB, AMJBS and EED.

For critical appraisal and assessment of the methodological quality of the included studies, the QUADAS 2 checklist was used (Quality Assessment of Diagnostic Accuracy Studies 2). Both risk of bias and concerns regarding applicability were checked [29].

The risk of bias was assessed for the following domains: patient selection, index test, reference test and patient flow. For each domain signaling questions were answered with yes, no, or unclear. With these answers, a risk of bias per domain was then assessed. The risk of bias was “low” if the answers to all questions were “yes.” If any of the questions was answered with “no,” a potential risk of bias exists, hence a judgment of “high.” The unclear category was used if insufficient data were reported to permit judgment.

Concerns regarding applicability were assessed for the following domains: patient selection, index test, reference test and patient flow.

Details on risk of bias and concerns regarding applicability are described in Table 1.

**Table 1:** QUADAS (Quality Assessment Tool for Diagnostic Accuracy Studies, version 2) by domain

Signaling questions and risk of bias	Patient selection	Signaling questions	Q1: Was a consecutive or random sample of patients enrolled?	YES/NO/UNCLEAR
			Q2: Was a case control design avoided?	YES/NO/UNCLEAR
			Q3: Did the study avoid inappropriate exclusions?	YES/NO/UNCLEAR
		Risk of bias	Could the selection of patients have introduced bias?^a^	LOW/HIGH/UNCLEAR
	Index test/MRI	Signaling questions	Q4: Were the MRI test results interpreted without knowledge of the results of the reference standard?	YES/NO/UNCLEAR
			Q5: If radiological criteria were used, were they (pro−/retrospectively) prespecified?	YES/NO/UNCLEAR
		Risk of bias	Could the conduct or interpretation of the MRI have introduced bias? ^a^	LOW/HIGH/UNCLEAR
	Reference tests	Signaling questions	Q6. Is the reference standard likely to correctly classify the target condition? ^b^	YES/NO/UNCLEAR
			Q7. Were the reference test results interpreted without knowledge of the results of the MRI?	YES/NO/UNCLEAR
		Risk of bias	Could the conduct or interpretation of the reference standard have introduced bias? ^a^	LOW/HIGH/UNCLEAR
	Flow and timing	Signaling questions	Q8. Was there an appropriate interval between WB-MRI and reference standard (<1 month for biopsy and any other imaging techniques and <12 months for FU)?	YES/NO/UNCLEAR
			Q9. Did all patients receive a reference standard?	YES/NO/UNCLEAR
			Q10. Were all patients included in the analysis (initially included based on inclusion and exclusion criteria)?	YES/NO/UNCLEAR
		Risk of bias	Could the patient flow have introduced bias? ^a^	LOW/HIGH/UNCLEAR
Concerns regarding applicability	Patient selection		Is there concern that the included patients do not match the review question? ^c^	LOW/HIGH
	Index test/MRI		Is there concern that the index test, its conduct or interpretation differs from the review question? ^d^	LOW/HIGH
	Reference tests		Is there concern that the reference test, its conduct or interpretation differs from the review question? ^e^	LOW/HIGH

^a^If answers to signaling questions (Q1-Q10) per domain are “YES” then risk of bias can be judged “LOW.” If any answer is “NO,” risk of bias can be judged “HIGH” (potential bias exists). If all answers are “UNCLEAR,” the risk of bias is also “UNCLEAR”

^b^In case of only biopsy and/or additional FU used

^c^Concern exists when 50% of patients included have other types of tumors than mentioned in inclusion criteria, or only adolescents or young adults are included

^d^Concern exists if the description of both conduction (magnetic field, coil, sequences) and interpretation (number and experience of observer, how data were compared with reference standard and MRI criteria for skeletal metastases) were either not described or were unclear

^e^Concern exists if MRI was also used in the reference standard

*FU* follow-up, *MRI* magnetic resonance imaging, *WB-MRI* whole-body magnetic resonance imaging

The following study design characteristics were recorded: a. First author, year of publication; b. Period of recruitment; c. Country of origin; d. Design of study (single center or multicenter; if authors from different institutions were involved regardless of patient inclusion, the design of the study was considered multicenter); e. Department of first author (Radiology or other), f. Data collection (Prospective or Retrospective or Unclear; in case informed consent was obtained, we considered the study to be prospective.) and g. Institutional Review Board approval (approved and informed consent obtained or approved and requirement for informed consent waived or not approved or unclear).

The recorded patient characteristics were: a. Inclusion and exclusion criteria; b. Number of patients included (either total number, included, or analyzed); c. Age (mean + standard deviation [SD] or median + range) in years, d. Male: female ratio and e. Distribution of patients (type of tumor).

Technical MRI features that were assessed: a. Magnetic field strength; b. Coil(s) used (only body coil or also other); c. Types of sequences (T1-weighted and/or T2-weighted and/or diffusion weighted imaging [DWI] and/or short tau inversion recovery [STIR] or other); d. Imaging planes (axial and/or coronal and/or sagittal), e. Examination time and f. Sedation.

Execution of MRI was considered to be described sufficiently if a-c were given.

Data on interpretation of WB-MRI that were recorded: a. Number of observers for interpretation; b. Experience of the observers; c. In case >1 observer, how data of the observers analyzed for comparison with reference standard were reported (consensus reading or separately/independently or unclear) and d. Criteria for skeletal metastases on WB-MRI. Interpretation of MRI was considered to be described sufficiently if a-d were given.

All reference tests used for comparison were recorded for each study. In addition, the time interval between WB-MRI and reference standard and any patients who were initially included (based on inclusion and exclusion criteria) but who did not undergo reference standard or were excluded for other reasons were reported.

Data were extracted on per-lesion, per-region or per-patient basis. For each study, data were extracted/constructed for WB-MRI compared with the reference standard. Per lesion: numbers of true positive (TP), false-negative (FN) and false-positive (FP) results were extracted and per-lesion sensitivity was calculated as follows: TP/(TP+FN); and positive predictive value (PPV) as follows: TP/(TP+FP).

Per region and per patient: numbers of true positive (TP), false-negative (FN), false-positive (FP) and true negative (TN) results were extracted and both sensitivity (TP/[TP+FN]) and specificity (TN/[TN+FP]) were calculated.

If more than one dataset was given (e.g., more observers or more comparisons), all datasets were extracted.

### Data analysis

Since the number of studies were limited and data was heterogeneous [30], meta-analysis with random-effect approach [31, 32] would not be suitable for pooling sensitivity, specificity or positive predictive values (PPV). All data are therefore presented per study.

## Results

### Search and selection of relevant studies

**Fig. 1:**
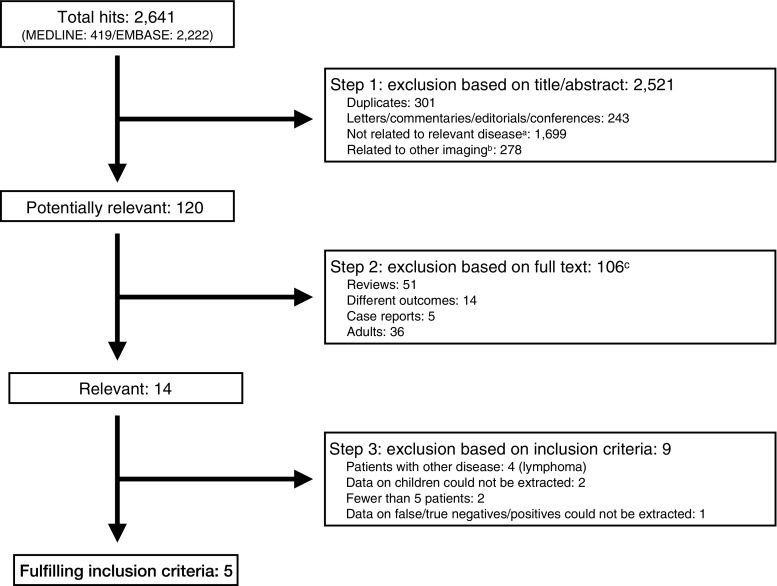
Search, selection and inclusion of relevant papers.^a^ Not relevant disease related (other disease, ovarian cancer, neurofibromatosis, cervix cancer, glioma).^b^ Other type of imaging was evaluated: such as PET, scintigraphy, CT. ^c^potentially relevant studies were checked on two occasions two weeks apart. TP=true positive; TN=true negative; FN=false negative

The searches in Medline and EMBASE databases resulted in 2,641 articles (Fig. 1). Based on title and abstract, 2,521 articles were excluded. The remaining 120 articles were double-checked on full text and an additional 106 articles were excluded as these were reviews (*n*=51), reported other outcome (*n*=14), case reports (*n*=5) and studies including a majority of adults (*n*=36). The remaining 14 articles were found to be relevant and were checked for fulfilling inclusion criteria.

### Inclusion of relevant studies

Of the 14 studies, 9 were excluded due to inclusion of patients with malignancies other than solid tumors (*n*=4, all patients had lymphoma) [33–36]; proportion of children unknown and/or data on children/adolescents could not be extracted (*n*=2) [37, 38]; fewer than 5 patients included with solid tumors (*n*=2) [39, 40] and data on true positives, false-negatives, false-positives and true negatives could not be extracted (*n*=1) [41].

Five studies [24–28] were included in this review. The complete search is presented in Fig. 1.

### Methodological quality characteristics

The risk of bias for patient selection was unclear in all studies for lack of information on whether patients were included consecutively or randomly (Q1) or whether patients were excluded inappropriately (Q3) (Table 2).

**Table 2:** Methodological quality criteria of the included studies: risk of bias and concerns regarding applicability

Author, year publication	Risk of bias ^a^														Concerns regarding applicability^b^		
	Patient selection				Index test: WB-MRI			Reference test			Flow and timing				Patient selection^c^	INDEX TEST: WB-MRI^d^	Reference test^e^
	Q1	Q2	Q3	Risk of bias ^a^	Q4	Q5	Risk of bias ^a^	Q6	Q7	Risk of bias ^a^	Q8	Q9	Q10	Risk of bias ^a^			
Daldrup-Link et al., 2001 [24]	Unclear	YES	Unclear	Unclear	YES	YES	Low	NO	Unclear	High	YES	YES	YES	Low	Low	High	High ^f^
Mazumdar et al., 2002 [26]	Unclear	YES	Unclear	Unclear	YES	YES	Low	NO	Unclear	High	YES	YES	YES	Low	Low	High	High ^g^
Goo et al., 2005 [25]	Unclear	YES	Unclear	Unclear	YES	YES	Low	NO	YES	High	YES	YES	YES	Low	High	Low	High
Kumar et al., 2008 [27]	Unclear	YES	Unclear	Unclear	YES	YES	Low	NO	Unclear	High	YES	YES	YES	Low	Low	High	High
Krohmer et al., 2010 [28]	Unclear	YES	Unclear	Unclear	YES	YES	Low	NO	YES	High	YES	YES	YES	Low	High	High	High

*Signaling questions per domain (Q1-Q10) are answered with YES/NO/UNCLEAR*

Patient selection: Q1. Was a consecutive or random sample of patients enrolled? Q2. Was a case control design avoided? Q3. Did the study avoid inappropriate exclusions?

Index test WB-MRI: Q4. Were the MRI test results interpreted without knowledge of the results of the reference standard? Q5. If radiological criteria were used, were they (pro−/retrospectively) pre-specified?

Reference test: Q6. Is the reference standard likely to correctly classify the target condition? Q7. Were the reference test results interpreted without knowledge of the results of the MRI?

Flow and timing: Q8. Was there an appropriate interval between WB-MRI and reference standard (< 1 month for biopsy and any other imaging techniques and < 12 months for FU)? Q9. Did all patients receive a reference standard? Q10. Were all patients included in the analysis (initially included based on inclusion and exclusion criteria)?

^a^ *If answers to signaling questions per domain are “YES” then risk of bias per domain can be judged “LOW”. If any answer is “NO” then risk of bias per domain can be judged “HIGH” (potential bias exists). If any answer is “UNCLEAR”, the risk of bias is also “UNCLEAR”*

Risk of bias per domain were*:*

Patient selection*:* Could the selection of patients have introduced bias?

Index test WB-MRI: Could the conduct or interpretation of the MRI have introduced bias?

Reference test: Could the conduct or interpretation of the reference standard have introduced bias?

Flow and timing: Could the patient flow have introduced bias?

^b^Concerns regarding applicability are answered with low/high

^c^Concern exists when more than 50% of patients included have other types of tumors than mentioned in inclusion criteria, or only adolescents or young adults are included

^d^Concern exists if the description of both conduction (magnetic field, coil, sequences) and interpretation (number and experience of observer, how data were compared with reference standard and the MRI criteria for skeletal metastases) were either not described or was unclear

^e^Concern exists if MRI was also used in the reference standard or if it was unclear whether MRI was part of the reference standard

^f^Not all lesions were histologically proven; all metastases underwent clinical and imaging follow up

^g^For the patients without proof of metastases, clinical and radiological follow-up documented the absence of disease

*MRI* magnetic resonance imaging, *WB-MRI* whole-body magnetic resonance imaging

The risk of bias for WB-MRI was low for all studies; this might be explained by the fact that all studies were initiated by the department of radiology and therefore both the blinded interpretation (Q4) and the radiologic criteria for skeletal metastases (Q5) were described in detail.

The risk of bias for the reference standard was high. None of the studies used only histology or follow-up as the reference standard; other imaging modalities and even MRI were included in the reference standard (Q6). In most studies, it was also unclear whether the reference test was interpreted without knowledge of the findings on WB-MRI (Q7) [24, 26, 27].

The risk of bias for flow and timing was low for all studies (Q8), all patients underwent a reference test (Q9) and all included patients were analyzed (Q10).

The concerns regarding applicability for patient selection were high in two studies [25, 28] (Table 2). In one study [28], more than 50% of the patients had tumors other than primary solid tumors. In the other study [25], more than 50% of patients were examined following the start of treatment.

The concerns regarding applicability for WB-MRI were high in four studies [24, 26–28] since no description on the experience of the radiologists was provided. If we discard the data on experience, most of the studies would have low concerns.

The concerns regarding applicability for the reference test were high in all studies, as MRI was included in the reference test or it was unclear if MRI was included in the reference test.

### Study design characteristics

All included studies were single center, prospectively designed by the department of radiology. All studies obtained informed consent. Studies were from Germany [24, 28], the United States [26], Korea [25] and India [27]. For 2 studies, the study period was unknown [24, 27]; the other studies were performed between March and December 2000 [26], May 2003 and September 2004 [25], and January 2004 and January 2006 [28].

### Patient characteristics

A total of 132 patients were included (age range: 4 months-19 years) (Table 3). Ninety-six patients had solid tumors, including 39 Ewing sarcomas/PNET (40.6%), 23 (ganglio)neuroblastomas (24%), 19 rhabdomyosarcomas (19.8%), 6 osteosarcomas (6.3%), 3 germ cell tumors (3.1%), 1 hepatoblastoma (1%), 1 Wilms tumor (1%), 1 melanoma (1%), 1 small round cell tumor (1%), 1 alveolar sarcoma (1%) and 1 fibrosarcoma (1%).

**Table 3:** Patient characteristics of the included studies

Authors, year of publication	Selection criteria (inclusion and/or exclusion criteria)	Total number of patients included in the article	Age (mean or median, and range)	Gender ratio (male: female)	Distribution of patients (type of tumor)
Daldrup-Link et al., 2001 [24]	Inclusion criteria: children and young adults with primary tumors that potentially metastasize to bone.	39	Mean age: 12.9 Range: 2–19 years	27:12	Ewing sarcoma, 20 Osteosarcoma, 3 Rhabdomyosarcoma, 3 Lymphoma, 2 Myelosarcoma, 1 Melanoma, 1 Langerhans cell histiocytosis, 9
Mazumdar et al., 2002 [26]	Inclusion criteria: small cell tumor in child, new or recurrent, MRI, reference imaging within 10 days Exclusion criteria: patients with newly diagnosed tumors who have had chemotherapy or radiation therapy for longer than 48 h before the imaging examinations, contraindications to sedation, a history of major allergic reaction to IV contrast material, and the presence of a cardiac pacemaker or intracranial vascular clips.	7 (5 newly diagnosed and 2 recurrences)	Mean age: 10.75 Range: 16–17 years	5:2	Ewing sarcoma, 2 (1 recurrent) Rhabdomyosarcoma, 4 (1 recurrent) Neuroblastoma, 1
Goo et al., 2005 [25]	Inclusion criteria: children who underwent WB-MRI and conventional oncological imaging within 15 days	36 (11 prior to chemotherapy, 25 after chemotherapy)	Median age: 3. 5 Range: 4–12 year	21:15	Rhabdomyosarcoma, 6 Lymphoma, 10 Neuroblastoma/ganglioneuroblastoma, 11/2 Germ-cell tumors, 3 Wilms tumor, 1 Hepatoblastoma, 1 PNET, 1 Small round cell neoplasm, 1
Kumar et al., 2008 [27]	Inclusion criteria: children and adolescents with histopathologically proven small-cell neoplasms who underwent WB-MRI, SSC and FDG PET/CT and iliac crest biopsy. Exclusion criteria: contraindications to sedation and the presence of a cardiac pacemaker or intracranial vascular clips	26	Range: 7–16 years	20:6	Ewing sarcoma/PNET, 11 Rhabdomyosarcoma, 5 Ganglioneuroblastoma, 1 Neuroblastoma, 8 Granulocytic sarcoma, 1
Krohmer et al., 2010 [28]	Inclusion criteria: children with suspicion or histological confirmation of malignant disease (lymphoma or solid malignant tumors); a maximum age of 18 years; at most 14 days between PET and WB-MRI, at least one conventional cross-sectional imaging examination, and no therapeutic action related to the malignant disease (e.g. chemotherapy). Exclusion criteria: patients without conventional cross-sectional imaging examinations and patients with suspicion or diagnosis of recurrent malignant disease	24	Mean age: 14 5/12 Range: 5–18 years	14:10	Hodgkin lymphoma, 11 Ewing sarcoma, 5 Osteosarcoma, 3 Follicular lymphoma, 1 Fibrosarcoma, 1 Rhabdomyosarcoma, 1 Alveolar sarcoma, 1 Langerhans cell histiocytosis, 1

*FDG* ^18^F–fluorodeoxyglucose, *MRI* magnetic resonance imaging, *PET* positron emission tomography, *PNET* primitive neuroectodermal tumour, *SSC* xxx, *WB-MRI* whole-body magnetic resonance imaging

Goo et al. [25] included a large number (25/36) of patients who had already been treated with chemotherapy and xMazumdar et al. [26] included two patients with a recurrence.

### Technical characteristics regarding whole-body magnetic resonance imaging

All scans were performed on a 1.5-T scanner (Table 4). Different types of coils were used depending on patient size: Young children were examined with head coils, older children with body-, phased array- and/or spine coils. Kumar et al. [27] used a combination of coil elements for the older children.

**Table 4:** Magnetic resonance imaging (MRI) technical features and interpretation of the included studies

Author, year publication	Magnetic field (T)	Coil	Sequences	Sedation	Examination time (min)	Imaging planes	Description of MRI technique^a^	Observers (number and experience defined and data analysis)	MRI criteria for skeletal metastasis	Interpretation described in detail^c^
Daldrup-Link et al., 2001 [24]	1.5	Head coil (small children) Body coil (older children) Spine coil for the spine	T1-W SE	No information	45 in young children, 55–60 in older children/ adolescents	AX, SAG, COR	Yes	2 observers, experience not defined, consensus reading	On T1-W SE: metastatic bone or bone marrow lesion defined as focal or diffuse hypointense signal relative to adjacent (or, in the extremities, contralateral) normal bone marrow.	No
Mazumdar et al., 2002 [26]	1.5	Head coil (small children), Body or phase array coil (older children)	COR T1 turbo SE, COR T2 turbo STIR	No^b^	Range: 15–20	COR	Yes	2 observers, experience not defined, consensus reading	On turbo STIR: skeletal metastasis defined as focal or diffuse hyperintensity of bone marrow relative to skeletal muscle or as destruction of cortical bone. On T1-W: skeletal metastases defined as areas of hypointensity	No
Goo et al., 2005 [25]	1.5	Body coil	COR turbo STIR (in all patients), SAG FS T2 (51/95 exams), SAG turbo STIR (32/95), COR FS T1 pre-and post-contrast (12/95)	Yes	Approximately 30	COR, SAG	Yes	2 observers, 1 year and 2 years of experience with WB-MRI, consensus reading	Bone marrow lesion hyperintense to muscle and normal bone marrow	Yes
Kumar et al., 2008 [27]	1.5	Total imaging matrix coil	SAG T1 of the spine, COR STIR	Yes	Mean: 50 (range 40–60)	COR, SAG	Yes	2 observers, experience not defined, consensus reading	On turbo STIR: skeletal metastasis defined as focal or diffuse hyperintensity of marrow, ≥signal intensity of CSF; focal or heterogeneous marrow signal variations or destruction of cortical bone. On T1-W: bone marrow metastases defined as areas of hypointensity, ≤signal intensity of skeletal muscle or as heterogeneous bone marrow signal variations.	No
Krohmer et al. 2010 [28]	1.5	Body coil	AX T2-STIR, COR T2-STIR, COR T1-TSE	Yes	Mean: 45	AX, COR	Yes	2 observers, experience not defined, consensus reading	Bone lesion: signal alteration (hyperintensity on T2-W)	No

^a^The execution of the MRI was described in sufficient detail if magnetic field, coil(s), and sequences were described

^b^WB-MRI was appended to another diagnostic test under sedation

^c^The interpretation of the MRI was described in sufficient detail on number and experience of observers and the criteria for skeletal metastases were given

*AX* axial, *COR* coronal, *CSF* cerebrospinal fluid, *FS* fat saturated, *SAG* sagittal, *STIR* short tau inversion recovery, *SE* spin echo, *TSE* turbo spin echo, *w* weighted

In all but one [24] study, a short tau inversion recovery (STIR) sequence was performed. In three studies, a subset of patients (young and/or uncooperative) were sedated for the WB-MRI-scan [25, 27, 28]; one study did not provide information on sedation [24] and in another study [26], WB-MRI was appended to another diagnostic test under sedation. The examination time varied from 15 to 60 min. None of the studies evaluated WB-DWI.

### Interpretation of whole-body magnetic resonance imaging

In all studies data, were interpreted by two radiologists in consensus (Table 4). On T1-weighted images, a metastatic bone or bone marrow lesion was defined as focal or diffuse hypointense bone marrow signal intensity relative to adjacent (or, in the extremities, contralateral) normal bone marrow. On STIR images, it was defined as focal or diffuse hyperintensity of bone marrow relative to skeletal muscle or as destruction of cortical bone.

### Reference test and flow of timing

All patients underwent a reference standard. Different reference standards were used, including MRI in three studies [25, 27, 28] (Table 5). The other two studies used clinical and imaging/radiologic follow-up without specifying which imaging modality was used [24, 26]. The time interval was less than 5 weeks and in studies without follow-up [26, 28] the interval between WB-MRI and reference standard was less than 13 days.

**Table 5:** Reference standard, and time interval between whole-body magnetic resonance imaging (WB-MRI) and reference standard

Study authors, year publication	Composition of reference standard	Interval between WB-MRI and reference standard	Proportion of study group undergoing reference standard
Daldrup-Link et al., 2001 [24]	Pathology (biopsy), PET, bone scintigraphy, clinical and imaging follow-up	Mean: 11 days, Range: 3–25 days maximum interval for imaging	39/39
Mazumdar et al., 2002 [26]	Bone scintigraphy, chest and abdominal CT, iliac crest biopsy (some patients), PET, histology, clinical and radiologic follow-up	<10 days	7/7
Goo et al., 2005 [25]	Pathology and other clinical results, including ultrasound, CT, MRI, scintigraphy, and clinical follow-up	≤15 days	36/36
Kumar et al., 2008 [27]	Bone scintigraphy, PET-CT, follow-up, dedicated MRI, iliac crest biopsy	≤25 days	26/26
Krohmer et al. 2010 [28]	Different cross-sectional imaging (MRI, CT and/or US)	Mean: 5 days Range: 0–13 days	24/24

*CT* computed tomography, *PET* positron emission tomography

### Data on per-lesion, per-region and per-patient

Data were reported on a per-lesion basis in three studies [24, 25, 28], per-region basis in one study [27] and per-patient basis in two studies [24, 26] (Table 6).

**Table 6:** Data on detection of skeletal lesions with whole-body magnetic resonance imaging

Study authors, year publication	Positives		Negatives		Sensitivity	PPV^a^	Specificity^b^
	TP	FP	FN	TN	TP/(TP+FN)	TP/(TP+FP)	TN/(TN+FP)
Per lesion							
Daldrup-Link et al., 2001 [24] (*n*=51 lesions)	42	3	9	NA	82.4%	93.3%	
Goo et al., 2005 [25] (*n*=112 lesions compared with bone scintigraphy)^c^	111	7	1	NA	99.1%	94.1%	
Goo et al., 2005 [25] (*n*=4 lesions compared with MIBG) ^c^	4	48	0	NA	100%	7.7%	
Goo et al., 2005 [25] (*n*=76 lesions compared with CT) ^c^	76	35	0	NA	100%	68.5%	
Krohmer et al., 2010 [28] (*n*=42 lesions)	42	NA^d^	0	NA	100%		
Per region							
Kumar et al., 2008 [27] (*n*=208 regions)	39	1	1	167	97.5%		99.4%
Per patient							
Daldrup-Link, et al., 2001 [24] (*n*=39 patients)	16	0	5	18	76.2%		100%
Mazumdar et al., 2002 [26] (*n*=7 patients)	2	0	0	5	100%		100%

^a^On per-lesion basis, positive predictive value is given

^b^On per-region and per patient basis, specificity is given

^c^In the study of Goo et al., different datasets were given

^d^88 lesions were only detected with MRI and not with the other modalities; however, these were both skeletal and extraskeletal lesions. The number of skeletal false-positives was not given

*CT* computed tomography, *FN* false-negative, *FP* false-positive, *MIBG* iodine-123-meta-iodobenzylguanidine scintigraphy, *NA* not applicable, *PPV* positive predictive value, *TP* true positive, *TN* true negative

Daldrup-Link et al. [24] described 51 bone lesions in 21 patients, proven by follow-up imaging or biopsy. WB-MRI detected 42 of these lesions (sensitivity: 82.4%); however, 9 lesions were false-negative on WB-MRI.

Goo et al. [25] presented three datasets, i.e. subsets for comparison of WB-MRI with bone scintigraphy (*n*=58), MIBG scan (*n*=26) and CT scan (*n*=48). For all subsets, a high sensitivity of WB-MRI was found (range: 99.1–100%). There was only one false-negative finding, occurring in the subset comparing WB-MRI with bone scintigraphy. In the other two datasets, the numbers of false-positives were high, especially in the subset of patients who underwent WB-MRI and MIBG scan (PPV=7.7%).

In the study by Krohmer et al. [28], WB-MRI had a high sensitivity (100%) but also a high number of false-positives (88/130): These lesions were both skeletal and extraskeletal lesions. The number of skeletal false-positives was not given.

Kumar et al. [27] evaluated bone metastases in 208 regions. According to the reference standard, 40 regions showed bone (marrow) lesions; WB-MRI detected metastases in 39 of these regions, resulting in a sensitivity of 97.5%. The specificity of WB-MRI was also high (99.4%).

In the study by Daldrup-Link et al. [24], 21 of 39 patients showed bone metastases and WB-MRI detected metastases in 16 of these patients, with a sensitivity of 76.2% and a specificity of 100%.

In the study by Mazumdar et al. [26], two patients had skeletal metastases, which were correctly detected on WB-MRI (sensitivity and specificity were both 100%).

## Discussion

### Summary of findings

With this systematic review, we provide a summary of the available evidence on the diagnostic performance of WB-MRI for detecting skeletal metastases in children with a malignant primary solid tumor. Because all included studies had a reference standard with a high risk of bias, it is difficult to determine the true value of WB-MRI.

The sensitivity of WB-MRI for detecting bone metastases per lesion ranged from 82.4% to 100%. The sensitivity upper range of 100% most likely is an overestimation of the sensitivity of MRI by incorporation bias because MRI was part of the reference standard in 3/5 studies [25, 27, 28].

In the study by Daldrup-Link et al. [24], the false-negative rate of WB-MRI was higher compared to the other studies. The reason for this lower sensitivity of MRI is unclear, but it may be due to the use of a T1-weighted sequence, instead of a STIR sequence in the other studies, although the authors state that T1-weighted images showed fewer movement artifacts, had a higher spatial resolution, and had a comparable sensitivity but higher specificity than STIR images in children. The false-negatives in their study were generally small lesions in small or flat bones (*n*=7/9).

A few studies found a high number of false-positives for WB-MRI. These high rates of false-positives might be explained by the reference tests used: MIBG and CT [25]. These reference tests might be less sensitive than WB-MRI, leading to a high false-positive rate (for lesions that might be true positives). In the study comparing WB-MRI with MIBG [25], the high false-positive rate of WB-MRI might be explained by the low specificity of the STIR sequence, since hyperintense lesions represent an increase in water content and may also be of traumatic, infectious or inflammatory origin or represent areas of highly cellular hematopoietic marrow, cysts or other benign lesions [30]. Furthermore, a substantial number of patients in this study were examined after treatment and osteomedullary metastases may continue to be visible on WB-MRI when they have become fibrotic [42]. Krohmer et al. [28] also described a high number of false-positive lesions on WB-MRI, but they did not mention how many of the lesions were in the skeleton. They also described 33 of the 88 false-positive lesions as bone lesions that had been detected in the peripheral skeleton of only 2 patients in whom conventional cross-sectional imaging of these particular areas had not been performed.

Finally, it should be pointed out that in all studies data were interpreted by two radiologists in consensus, which is a known study limitation [43].

### Strengths of this review

In the past 20 years, several studies have been conducted to study the diagnostic value of WB-MRI for detecting skeletal metastases in solid tumors [18–20]. A recent study by Kembhavi et al. [37] showed that WB-MRI using the STIR sequence detected all bone lesions both in adults and children.

However, differences between adults and children are particularly important in this context. The types of tumors children develop, and the biology of these types of tumors, generally differ from adult types. Furthermore, it is during childhood and adolescence that bone marrow conversion takes place, from predominantly hematopoietic (red marrow) at birth to fatty (yellow) marrow and this process complicates the interpretation of bone marrow lesions in the pediatric age group. This enhances the importance of summarizing the existing evidence on the diagnostic value of WB-MRI for detecting skeletal metastases specifically for children and adolescents.

### Limitations of this review

One of the limitations of this review is the low number of included studies. Although there were a significant number of relevant studies, many studies included both adults and children, from which the data of children could not be extracted.

Another limitation is related to the reference standard: the lack of histopathological proof of many lesions and the fact that various different imaging modalities were used as reference standard, including MRI. However, all included patients did undergo a reference standard.

Furthermore, the included patient cohorts were rather heterogeneous: All studies pooled patients with different types of malignancies, and in most studies results were not specified per tumor type. Hence, no evidence per specific tumor type on the value of WB-MRI in the staging process could be extracted. Nevertheless, we can conclude that, in general, WB-MRI seems to be a sensitive tool for detecting skeletal metastatic lesions.

Finally, all studies have either an unclear or high risk of bias or high concern on applicability, although in some aspects, our criteria might have been too strict: We judged low for lack of information on experience of the observers, but if we discard these data, most of the studies would have low concerns.

## Conclusion

WB-MRI seems a promising radiation-free alternative to the currently used modalities for detecting skeletal metastases in children with solid tumors. The heterogeneity and the small size of the studies, however, do not provide conclusive evidence that WB-MRI can offer equally good or better staging for each specific tumor type. In addition, before implementing WB-MRI in the staging process, the choice of sequence(s) and parameters should be standardized.

## Electronic supplementary material

ESM 1 (DOC 24 kb)

## References

[1] Maris JM (2010). Recent advances in neuroblastoma. N Engl J Med.

[2] DuBois SG, Kalika Y, Lukens JN (1999). Metastatic sites in stage IV and IVS neuroblastoma correlate with age, tumor biology, and survival. J Pediatr Hematol Oncol.

[3] Weiss AR, Lyden ER, Anderson JR (2013). Histologic and clinical characteristics can guide staging evaluations for children and adolescents with rhabdomyosarcoma: a report from the Children's oncology group soft tissue sarcoma committee. J Clin Oncol.

[4] Cotterill SJ, Ahrens S, Paulussen M (2000). Prognostic factors in Ewing's tumor of bone: analysis of 975 patients from the European intergroup cooperative Ewing's sarcoma study group. J Clin Oncol.

[5] Kager L, Zoubek A, Potschger U (2003). Primary metastatic osteosarcoma: presentation and outcome of patients treated on neoadjuvant cooperative osteosarcoma study group protocols. J Clin Oncol.

[6] Kager L, Zoubek A, Kastner U (2006). Skip metastases in osteosarcoma: experience of the cooperative osteosarcoma study group. J Clin Oncol.

[7] Marsden HB, Lawler W (1978). Bone-metastasizing renal tumour of childhood. Br J Cancer.

[8] Uslu L, Donig J, Link M (2015). Value of 18F-FDG PET and PET/CT for evaluation of pediatric malignancies. J Nucl Med.

[9] Sharp SE, Trout AT, Weiss BD (2016). MIBG in neuroblastoma diagnostic imaging and therapy. Radiographics.

[10] Robbins E (2008). Radiation risks from imaging studies in children with cancer. Pediatr Blood Cancer.

[11] Harrison DJ, Parisi MT, Shulkin BL (2017). The role of 18F-FDG-PET/CT in pediatric sarcoma. Semin Nucl Med.

[12] Pearce MS, Salotti JA, Little MP (2012). Radiation exposure from CT scans in childhood and subsequent risk of leukaemia and brain tumours: a retrospective cohort study. Lancet.

[13] Hall EJ (2002). Lessons we have learned from our children: cancer risks from diagnostic radiology. Pediatr Radiol.

[14] Raissaki M, Demetriou S, Spanakis K (2017). Multifocal bone and bone marrow lesions in children - MRI findings. Pediatr Radiol.

[15] Hargaden G, O'Connell M, Kavanagh E (2003). Current concepts in whole-body imaging using turbo short tau inversion recovery MR imaging. AJR Am J Roentgenol.

[16] Lauenstein TC, Goehde SC, Herborn CU (2004). Whole-body MR imaging: evaluation of patients for metastases. Radiology.

[17] Eustace S, Tello R, DeCarvalho V (1997). A comparison of whole-body turboSTIR MR imaging and planar 99mTc-methylene diphosphonate scintigraphy in the examination of patients with suspected skeletal metastases. AJR Am J Roentgenol.

[18] Schmidt GP, Reiser MF, Baur-Melnyk A (2009). Whole-body MRI for the staging and follow-up of patients with metastasis. Eur J Radiol.

[19] Frat A, Agildere M, Gencoglu A (2006). Value of whole-body turbo short tau inversion recovery magnetic resonance imaging with panoramic table for detecting bone metastases: comparison with 99MTc-methylene diphosphonate scintigraphy. J Comput Assist Tomogr.

[20] Walker R, Kessar P, Blanchard R (2000). Turbo STIR magnetic resonance imaging as a whole-body screening tool for metastases in patients with breast carcinoma: preliminary clinical experience. J Magn Reson Imaging.

[21] Ghanem N, Uhl M, Brink I (2005). Diagnostic value of MRI in comparison to scintigraphy, PET, MS-CT and PET/CT for the detection of metastases of bone. Eur J Radiol.

[22] Chan BY, Gill KG, Rebsamen SL (2016). MR imaging of pediatric bone marrow. Radiographics.

[23] Burdiles A, Babyn PS (2009) Pediatric bone marrow MR imaging. Magn Reson Imaging Clin N Am 17:391–409, v 10.1016/j.mric.2009.03.001 19524192

[24] Daldrup-Link HE, Franzius C, Link TM (2001). Whole-body MR imaging for detection of bone metastases in children and young adults: comparison with skeletal scintigraphy and FDG PET. AJR Am J Roentgenol.

[25] Goo HW, Choi SH, Ghim T (2005). Whole-body MRI of paediatric malignant tumours: comparison with conventional oncological imaging methods. Pediatr Radiol.

[26] Mazumdar A, Siegel MJ, Narra V (2002). Whole-body fast inversion recovery MR imaging of small cell neoplasms in pediatric patients: a pilot study. AJR Am J Roentgenol.

[27] Kumar J, Seith A, Kumar A (2008). Whole-body MR imaging with the use of parallel imaging for detection of skeletal metastases in pediatric patients with small-cell neoplasms: comparison with skeletal scintigraphy and FDG PET/CT. Pediatr Radiol.

[28] Krohmer S, Sorge I, Krausse A (2010). Whole-body MRI for primary evaluation of malignant disease in children. Eur J Radiol.

[29] Whiting PF, Rutjes AW, Westwood ME (2011). QUADAS-2: a revised tool for the quality assessment of diagnostic accuracy studies. Ann Intern Med.

[30] Higgins JP, Thompson SG, Deeks JJ (2003). Measuring inconsistency in meta-analyses. BMJ.

[31] Reitsma JB, Glas AS, Rutjes AW (2005). Bivariate analysis of sensitivity and specificity produces informative summary measures in diagnostic reviews. J Clin Epidemiol.

[32] Leeflang MM, Deeks JJ, Rutjes AW (2012). Bivariate meta-analysis of predictive values of diagnostic tests can be an alternative to bivariate meta-analysis of sensitivity and specificity. J Clin Epidemiol.

[33] Kellenberger CJ, Miller SF, Khan M (2004). Initial experience with FSE STIR whole-body MR imaging for staging lymphoma in children. Eur Radiol.

[34] Punwani S, Cheung KK, Skipper N (2013). Dynamic contrast-enhanced MRI improves accuracy for detecting focal splenic involvement in children and adolescents with Hodgkin disease. Pediatr Radiol.

[35] Littooij AS, Kwee TC, Barber I (2014). Whole-body MRI for initial staging of paediatric lymphoma: prospective comparison to an FDG-PET/CT-based reference standard. Eur Radiol.

[36] Punwani S, Taylor SA, Bainbridge A (2010). Pediatric and adolescent lymphoma: comparison of whole-body STIR half-Fourier RARE MR imaging with an enhanced PET/CT reference for initial staging. Radiology.

[37] Kembhavi SA, Rangarajan V, Shah S (2014). Prospective observational study on diagnostic accuracy of whole-body MRI in solid small round cell tumours. Clin Radiol.

[38] Mentzel HJ, Kentouche K, Sauner D (2004). Comparison of whole-body STIR-MRI and 99mTc-methylene-diphosphonate scintigraphy in children with suspected multifocal bone lesions. Eur Radiol.

[39] Laffan EE, O'Connor R, Ryan SP (2004). Whole-body magnetic resonance imaging: a useful additional sequence in paediatric imaging. Pediatr Radiol.

[40] Klenk C, Gawande R, Uslu L (2014). Ionising radiation-free whole-body MRI versus (18)F-fluorodeoxyglucose PET/CT scans for children and young adults with cancer: a prospective, non-randomised, single-centre study. Lancet Oncol.

[41] Siegel MJ, Acharyya S, Hoffer FA (2013). Whole-body MR imaging for staging of malignant tumors in pediatric patients: results of the American College of Radiology Imaging Network 6660 trial. Radiology.

[42] Ollivier L, Gerber S, Vanel D (2006). Improving the interpretation of bone marrow imaging in cancer patients. Cancer Imaging.

[43] Bankier AA, Levine D, Halpern EF (2010). Consensus interpretation in imaging research: is there a better way?. Radiology.

