# Supplementary material for: Whole-body magnetic resonance imaging for detection of skeletal metastases in children and young people with primary solid tumors - systematic review
Source: Pediatr Radiol. 2017 Nov 18;48(2):241–52. doi: 10.1007/s00247-017-4013-8 (PMC5790860; doi:10.1007/s00247-017-4013-8)
Supplement: Supplementary file 1 — (DOC 24 kb) [file 247_2017_4013_MOESM1_ESM.doc]

**Supplement 1. Search strategy for the systematic review (up to 15 May 2017)**

| **MEDLINE** | **Search terms** |
| --- | --- |
| #1 | ((Whole-body OR whole body) AND ("Magnetic Resonance Imaging"[Mesh])) OR ("Diffusion Magnetic Resonance Imaging"[Mesh]) OR ("Whole Body Imaging"[Mesh]) |
| #2 | ("Medical Oncology"[Mesh]) OR ("Neoplasms"[Mesh]) OR ("Neoplasm Staging"[Mesh]) OR ("Neoplasm Metastasis"[Mesh]) |
| # 3 | ("Adolescent"[Mesh]) OR ("Child"[Mesh]) OR ("Child, Preschool"[Mesh]) OR ("Young Adult"[Mesh]): |
| #4 | # 1 AND #2 AND #3 |

| **EMBASE** | **Search terms** |
| --- | --- |
| #1 | (Whole body.mp or whole-body.mp) AND exp nuclear magnetic resonance imaging/: |
| #2 | Exp diffusion weighted imaging/: |
| #3 | Exp whole body imaging/: |
| #4 | #1 OR #2 Or #3: |
| #5 | Exp oncology/OR exp neoplasms/or exp cancer staging/OR exp metastasis/ |
| #6 | Adolescent/OR child/OR preschool child/ or exp. Young adults/ |
| #7 | #4 AND#5AND#6 |
